# Genome-wide association study of susceptibility loci for breast cancer in Sardinian population

**DOI:** 10.1186/s12885-015-1392-9

**Published:** 2015-05-10

**Authors:** Grazia Palomba, Angela Loi, Eleonora Porcu, Antonio Cossu, Ilenia Zara, Mario Budroni, Mariano Dei, Sandra Lai, Antonella Mulas, Nina Olmeo, Maria Teresa Ionta, Francesco Atzori, Gianmauro Cuccuru, Maristella Pitzalis, Magdalena Zoledziewska, Nazario Olla, Mario Lovicu, Marina Pisano, Gonçalo R. Abecasis, Manuela Uda, Francesco Tanda, Kyriaki Michailidou, Douglas F. Easton, Stephen J. Chanock, Robert N. Hoover, David J. Hunter, David Schlessinger, Serena Sanna, Laura Crisponi, Giuseppe Palmieri

**Affiliations:** 1 Istituto di Chimica Biomolecolare, Consiglio Nazionale delle Ricerche, Traversa La Crucca 3, Baldinca Li Punti, 07100 Sassari, Italy; 2 Istituto di Ricerca Genetica e Biomedica (IRGB), Consiglio Nazionale delle Ricerche (CNR), Monserrato, 09042 Cagliari, Italy; 3 Istituto di Anatomia Patologica, Azienda Ospedaliero Universitaria, Sassari, Italy; 4 Center for Advanced Studies, Research and Development in Sardina (CRS4), Pula, Cagliari, Italy; 5 Servizio di Epidemiologia, Azienda Sanitaria Locale n. 1, Sassari, Italy; 6 Servizio di Oncologia Medica, Azienda Sanitaria Locale n. 1, Sassari, Italy; 7 Dipartimento di Oncologia Medica, Azienda Ospedaliero Universitaria, Monserrato, Cagliari, Italy; 8 Center for Statistical Genetics, University of Michigan, Ann Arbor, MI USA; 9 Department of Public Health and Primary Care, Centre for Cancer Genetic Epidemiology, University of Cambridge, Cambridge, UK; 10 Department of Oncology, Centre for Cancer Genetic Epidemiology, University of Cambridge, Cambridge, UK; 11 Division of Cancer Epidemiology and Genetics, National Cancer Institute, Bethesda, MD USA; 12 Harvard School of Public Health, Boston, MA USA; 13 Laboratory of Genetics, National Institute on Aging, National Institutes of Health, Baltimore, MD USA; 14 Unit of Cancer Genetics, Institute of Biomolecular Chemistry (ICB), National Research Council (CNR), Traversa La Crucca 3, Baldinca Li Punti, 07100 Sassari, Italy

**Keywords:** Breast cancer risk, *BRCA1/2* mutation analysis, Genome-wide association study, Sardinian population

## Abstract

**Background:**

Despite progress in identifying genes associated with breast cancer, many more risk loci exist. Genome-wide association analyses in genetically-homogeneous populations, such as that of Sardinia (Italy), could represent an additional approach to detect low penetrance alleles.

**Methods:**

We performed a genome-wide association study comparing 1431 Sardinian patients with non-familial, *BRCA1/2*-mutation-negative breast cancer to 2171 healthy Sardinian blood donors. DNA was genotyped using GeneChip Human Mapping 500 K Arrays or Genome-Wide Human SNP Arrays 6.0. To increase genomic coverage, genotypes of additional SNPs were imputed using data from HapMap Phase II. After quality control filtering of genotype data, 1367 cases (9 men) and 1658 controls (1156 men) were analyzed on a total of 2,067,645 SNPs.

**Results:**

Overall, 33 genomic regions (67 candidate SNPs) were associated with breast cancer risk at the *p* < 10^−6^ level. Twenty of these regions contained defined genes, including one already associated with breast cancer risk: *TOX3*. With a lower threshold for preliminary significance to p < 10^−5^, we identified 11 additional SNPs in *FGFR2,* a well-established breast cancer-associated gene. Ten candidate SNPs were selected, excluding those already associated with breast cancer, for technical validation as well as replication in 1668 samples from the same population. Only SNP rs345299, located in intron 1 of *VAV3*, remained suggestively associated (*p*-value, 1.16x10^−5^), but it did not associate with breast cancer risk in pooled data from two large, mixed-population cohorts.

**Conclusions:**

This study indicated the role of *TOX3* and *FGFR2* as breast cancer susceptibility genes in *BRCA1/2*-wild-type breast cancer patients from Sardinian population.

**Electronic supplementary material:**

The online version of this article (doi:10.1186/s12885-015-1392-9) contains supplementary material, which is available to authorized users.

## Background

Breast cancer is the most common malignancy in women in western countries, currently accounting for one-third of all female cancer cases [1]. A family history of breast cancer is the principal risk factor for developing the disease [2]. Linkage studies in families have identified several high-penetrance mutations in *BRCA1, BRCA2* and other genes as causative of disease in 5 %-10 % of cases [3, 4]. Additionally, a combined approach of family-based and case–control studies revealed that mutations in several genes encoding proteins involved in DNA repair and functionally interacting with the BRCA1/2 proteins are associated with a moderate risk of breast cancer, contributing to another 10 %–15 % of cases [5]. Genome-wide association (GWA) studies have so far identified at least 72 common lower penetrance alleles associated with breast cancer [6, 7]. A large fraction of these susceptibility alleles are associated with increased risk in persons with a family history of breast cancer despite the absence of mutations in *BRCA1* or *BRCA2*, accounting for about 30 % of the familial risk of the disease [7]. Other alleles, such as those that map to the *FGFR2* and *TOX3* genes, act as risk modifiers in *BRCA1/2*-mutation carriers [3, 8, 9].

Results from a meta-analysis of GWA studies suggest that a substantial fraction of the residual familial aggregation cases can be explained by other common single nucleotide polymorphisms (SNPs) not yet identified [7]. In particular, the authors hypothesized that more than 1000 additional loci may be involved in breast cancer susceptibility. Because of their low penetrance and the small fraction of familial cases, it is unlikely that other susceptibility genes will be identified through additional family-based studies. A promising approach could be to conduct new studies in non-familial cases, such as case–control in populations with less genetic heterogeneity.

One population with notable genetic inter-relatedness is that of the Mediterranean island of Sardinia (Italy). In Sardinia, breast cancer represents the principal death-causing malignancy among women, with an incidence similar to that observed in other western populations [10]. The Sardinian population (1.67 million in 2010, according to the Italian National Institute of Statistics) is isolated, with considerable inter-relatedness and founder effects for several genetic diseases (e.g. thalassemia) [11, 12]. The relatively homogeneous genetic make-up of the Sardinian population offered an opportunity to search for genetic determinants of breast cancer, requiring fewer cases to establish association with a susceptibility locus than do mixed populations. We have conducted such a case–control GWA study for breast cancer risk in our collection of Sardinian breast cancer patients who are negative for *BRCA1* or *BRCA2* mutations.

## Methods

### Breast cancer cases and controls

From January 1998 to December 2006, we recruited 1698 patients with breast cancer from the four main oncology units in the Region of Sardinia (Azienda Ospedaliero Universitaria of Sassari, Azienda Sanitaria Locale of Sassari, Businco Oncologic Institute, and University of Cagliari). This cohort includes 1085 patients recruited in 1998-2003 [13]. Inclusion criteria were: (i) a histopathological diagnosis of any type of breast cancer, and (ii) self-reported Sardinian origin, defined as both biological parents and all four biological grandparents born on the island. No exclusion criteria were applied; in particular, patients were not selected for age, gender, grade or stage of cancer, or family history of any cancer.

Family history for cancer was evaluated through specific questionnaires during the follow-up visits at the different departments of the participating institutions. Cases were classified as non-familial when less than three (0, 1, or 2) affected members with breast or ovarian cancer were present in first- and second-degree relatives. A sample of peripheral blood was obtained for DNA extraction. Pathological TNM (tumor, node, metastasis) classification and immuno-histochemistry profile for estrogen receptors (ER), progesterone receptors (PR) and HER2 (receptor tyrosine-protein kinase erbB-2) were also obtained, when available.

**Fig. 1:**
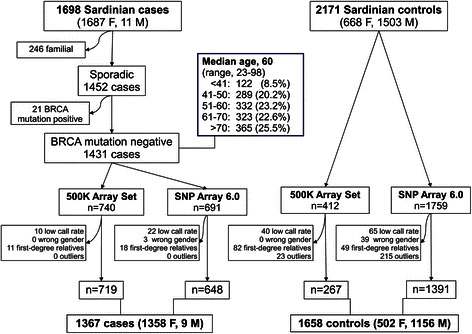
Flow chart of the selection of cases and controls throughout the study

Controls consisted of 2171 healthy persons recruited at community blood donation centers across the island and at the transfusion center of Azienda Ospedaliera Brotzu in Cagliari. Controls were included if at least three out of four grandparents were born in Sardinia and if they reported no type of cancer for their first-degree relatives. Overall, 1503 (69.2 %) were males, in line with the male preponderance among Italian blood donors [CENSIS at http://www.censis.it/] (Fig. 1). Since the present study was aimed at detecting low penetrance alleles at autosomic level, no significant differences were expected by the use of control males. In fact, allelic transmission is identical in males and females as the entire population should comply with the Hardy Weinberg law. As some randomness is expected, we specifically assessed our best SNPs for the impact of males in the controls set, excluding bias. This was added to a genome-wide check of allele frequency distribution between genders. Of note, the same approach of including males among controls has also been adopted by previous studies [14–16], all of them reporting a genome-wide check of differences between males and females, with no significant diversity in alleles’ distribution. In such latter studies, percentages of male controls were reported to up to 78 %, which is thus consistent with the frequency (69 %) reported in our series.

Both cases and controls gave written informed consent for their biological samples and clinical data to be used for research purposes. The study protocol was reviewed and approved by the Ethics Committee of the ASL8 Cagliari and the Bioethics Committee of the Sassari Healthcare District.

### BRCA mutation analysis

From our cohort of 1698 breast cancer patients, we selected 1452 non-familial cases who were then tested for *BRCA1* and *BRCA2* germline mutations. The entire coding sequences and intron-exon boundaries of the *BRCA1* and *BRCA2* genes were screened by denaturing high-performance liquid chromatography (DHPLC) followed by direct sequencing on an automated DNA sequencer (ABI Prism 3100 Genetic Analyzer, Applied Biosystems, Foster City, USA). Protocols for PCR-based amplification and mutation analysis of exons and exon-intron boundaries were as previously reported [17]. Familial cases (N = 246) were excluded on the basis of the presence of at least three family members (the proband and at least two other first- or second-degree relatives) having with either breast or ovarian cancer. Overall, 21 cases (1.4 %) had a mutation in *BRCA1* or *BRCA2* and were excluded, leaving 1431 cases for the GWA study. The median age of the 1431 cases at diagnosis was 60 (range, 23–98); 11 cases (0.8 %) were male (Fig. 1).

### Genotyping and quality controls

DNA was isolated from peripheral blood and stored at -80 °C. Genotyping was performed at the Institute of Genetic and Biomedical Research using Affymetrix technology according to the manufacturer’s protocols. At first, 740 cases and 412 controls were genotyped using the GeneChip Human Mapping 500 K Array Set (analyzed from 2005 to 2006). The rest, 691 cases and 1759 controls, were assayed in the same center with the newer Genome-Wide Human SNP Array 6.0 (analyzed from 2007 to 2009) (Fig. 1).

Genotypes for individuals assessed with the 500 K Array and the 6.0 array set were called respectively with the BRLMM algorithm and with Birdseed v2 [18]. The latter algorithm was applied to a unique cluster containing all cases and controls, given its sensitivity to plate bias. A methodological limitation of the current study came from the use of two different microarray genotyping platforms. This choice was due to the adoption of the larger Array 6.0 (permitting the testing of 900 K SNPs) when it became available, but it was not feasible to retest the initial 740 cases and 412 controls with the larger panel. As a result, we analyzed directly only those SNPs that were represented on both platforms (240742 SNPs). Individuals with a SNP call rate <90 % were excluded from the analysis, as were individuals whose recorded gender was different from that predicted by the genetic data.

SNPs showing significant (*p* < 1x10^−6^) deviation from Hardy-Weinberg equilibrium in controls, minor allele frequency (MAF) <5 % or sample call rate <95 %, were filtered out. Moreover, for SNPs tested on both platforms, those with allele frequencies differing by >10 % in controls were excluded. We then left out all SNPs that were not represented on both platforms. Finally, to ensure that the dataset contained only unrelated persons, we used RELPAIR software [19] to estimate genotype sharing between all possible pairs of individuals based on a subset of 10000 quality-checked SNPs. When two persons were found to be first-degree relatives, we excluded the one with lower call rate, except when the pair consisted of one case and one control, in which we excluded the control.

To avoid bias introduced by population stratification, we performed principal component analysis (PCA) using Eigensoft 3.0 software [20, 21]. Individuals flagged as outliers in the PCA analyses (>6 standard deviations from the mean) were excluded. The principal component eigenvectors for the remaining individuals were recalculated and the axes were then used as covariates to calculate adjusted *p* values for association with breast cancer; the genomic control parameter was 1.149 (Additional file 1: Figure S1). For all SNPs with adjusted *p* < 10^−6^, we visually inspected the discrimination plots and kept only those with good plots (three distinct data clusters). A PCA supporting the overall homogeneity of our Sardinian sample in respect to general Europeans is showed in Additional file 2: Figure S2.

### Imputation and identification of candidate SNPs

To improve coverage of the genome, we increased the set of SNPs tested for association through imputation. MACH software (version 1.0) was used to impute non-genotyped markers based on the phased haplotypes from HapMap Phase II (CEU, release 22) and the set of 270742 quality-controlled markers represented on both platforms. Imputation increased the SNP coverage to a total of 2067645 markers (Additional file 3: Table S1), though without direct scoring of SNPs that might have had higher *p*-values.

After imputation, we considered only markers with MAF > 1 % and imputation quality (RSQR) >0.3 (RSQR infers r^2^ between true and estimated allele counts [22]). These markers were assessed with a likelihood ratio test to identify those with additive effects on modifying the risk of breast cancer; this test was implemented in mach2dat, in the MACH package, using allele dosages and the estimated eigenvectors as covariates in the model. We selected SNPs with *p* < 1x10^−6^ and examined the discrimination plots of all the other genotyped SNPs in the surrounding 200 kb genomic region. SNPs residing in genomic regions where all other genotyped SNPs had good discrimination plots were considered candidate markers.

### Validation of candidate markers

Validation of the SNPs classified as candidate markers, was done by regenotyping them with custom TaqMan SNP genotyping assays (Life Technologies). This validation step was done using DNA from a subset of the Sardinian cases (1362) and controls (1514) in this study who passed quality control filtering at the sample level in microarray genotyping (SNP call rate >90 % and no error in gender determination).

We attempted to replicate promising signals in a wider set of DNAs consisting in additional 201 Sardinian cases and 1467 controls (630 females and 1038 males) collected after December 2006 within the same study protocol and with the same features as the original set.

Replication analyses were performed using data from a combined analyses of eleven GWAS in other populations of European ancestry, comprising 16,195 cases and 18,980 controls, and data from 45,290 cases and 41,880 controls of European ancestry from 41 studies collaborating in the Breast Cancer Association Consortium (BCAC), which were genotyped with a custom array (iCOGS) ([7]; http://gameon.dfci.harvard.edu/gameon).

For all studies except BCFR, BPC3 and TNBCC, genotypes were estimated by imputation, using IMPUTE2 [23] and the 1000 genomes March 2012 release as a reference panel, after prephasing with SHAPEIT [24]. Per-allele odds ratios (ORs) and standard errors for individual studies were generated using SNPTEST [25]. BCFR, BPC3 and TNBCC performed imputation using MACH and Minimac. Estimated ORs for the combined analysis were generated using a fixed-effect meta-analysis, using METAL [22]. For the combined analysis of the GWAS and iCOGS, we reanalyzed the iCOGS data to remove samples also included in a GWAS, to generate independent datasets.

### Genotype associations with clinical data

Chi-square and Fisher’s exact tests were used to evaluate possible associations between tumor phenotype (ER, PR, HER2, pT, pN, M) and the genotypes of candidate SNPs. Statistical tests were performed using SPSS statistical software, version 15.0. All tests were two-tailed and a *p* < 0.05 indicated significance.

### Evaluation of GWAS-identified breast cancer risk variants in our Sardinian cohort

GWA studies have so far identified at least 72 common lower penetrance alleles associated with a mild increase in the risk of breast cancer [6, 7]. We evaluated index SNPs (when not available, proxies) in all 72 breast cancer susceptibility loci identified to date in our subset cohort (1367 cases and 1658 controls).

## Results

To search for new loci associated with breast cancer risk, we genotyped germline DNA of 1431 Sardinian patients with sporadic breast cancer (*BRCA*-mutation-negative) and a set of 2171 healthy blood donor controls. After quality control filtering of genotype data at the sample level, 1367 cases and 1658 controls were analyzed. For 921 cases (67 %), we had data regarding TNM classification and receptor status (Table 1). Among the 775 patients tested for all three receptors, the predominant molecular subtype was ER+/PR+/HER2-, found in 70.3 % of cases. Moreover, the percentage of triple-negative (ER-/PR-/HER2-) cases was low, 7.2 %.

**Table 1:** Tumor characteristics at the time of diagnosis, for 921 patients (all women) with sporadic, BRCA-mutation-negative breast cancer

Characteristic	Patients, n (%)
Pathological TNM classification	
pT1-2	830 (90.1)
pT3-4	91 (9.9)
pN0	533 (57.9)
pN1	388 (42.1)
M0	751 (81.5)
M1	43 (4.7)
Mx	127 (13.8)
Receptor status	
ER-	234 (25.4)
ER+	687 (74.6)
PR-	260 (28.2)
PR+	661 (71.8)
HER2-	601 (65.3)
HER2+	174 (18.9)
Not tested	146 (15.8)
Molecular subtype^a^	
ER+/PR+/HER2-	545 (70.3)
ER+/PR+/HER2+	142 (18.3)
ER-/PR-/HER2+	32 (4.1)
Triple negative (ER-/PR-/HER2-)	56 (7.2)

ER, estrogen receptor; PR, progesterone receptor.

^a^ For the 775 cases tested for all three receptor markers.

**Fig. 2:**
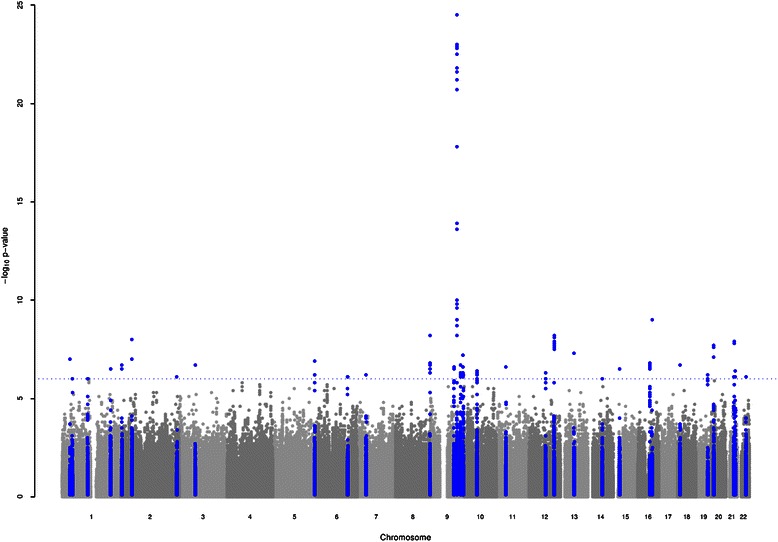
Manhattan plot for this genome-wide association study of sporadic breast cancer in Sardinian population. Data shown are the negative logarithm of the association *p-*value for each single nucleotide polymorphism. The horizontal line indicates the significance cut-off at *p* < 10^−6^

The genome wide analysis conducted on a set of 2067645 markers (Methods and Additional file 4: Table S6), revealed 33 genomic regions on 20 chromosomes that were suggestively associated with breast cancer risk at *p* < 1x10^−6^ (Fig. 2). In particular, 7 regions reached the genome wide significance threshold (*p* = 5x10^−8^; Table 2). The 33 suggestive genomic regions contained a total of 67 SNPs with *p* < 10^−6^ (Table 2, Additional file 3: Table S2).

**Table 2:** Most significant SNPs in 33 genomic regions associating at the *p* < 10^−6^ level with sporadic, *BRCA*-mutation-negative breast cancer cases in the Sardinian population

Chromosomal region	SNP	Position	Alleles	Frequency		RSQR	OR (CI 95%)	*p*	Gene
				Cases	Controls				
1p35.1	rs9425977	34238042	C/T	0.082	0.059	0.379	2.597 (1.817 - 3.709)	9.51E-08	*CSMD2*
1p34.1	rs2477618	44271050	C/G	0.077	0.058	0.416	2.312 (1.647 -3.245)	9.13E-07	*-*
1p13.3	rs345299	108249656	A/C	0.465	0.534	0.957	0.755 (0.676 - 0.845)	9.40E-07	*VAV3*
1q32.1	rs6661074	203605742	A/G	0.483	0.425	0.731	1.408 (1.235 - 1.605)	3.10E-07	*LEMD1*
2p25.3	rs13393791	3239073	C/T	0.074	0.039	0.989	1.862 (1.469 - 2.361)	1.99E-07	*TSSC1*
2p21	rs17032957	45134208	A/G	0.701	0.747	0.462	0.597 (0.500 - 0.712)	9.17E-09	*SIX2/SIX3*
2q37.1	rs838436	232291757	A/G	0.908	0.926	0.308	0.415 (0.291 - 0.592)	8.45E-07	*-*
3p14.1	rs9810816	67158548	C/G	0.935	0.955	0.404	0.368 (0.251 - 0.541)	1.86E-07	*-*
5q35.2	rs17076993	173564400	C/T	0.071	0.049	0.447	2.507 (1.768 - 3.553)	1.29E-07	*-*
6q23.1	rs3777428	130495535	C/T	0.926	0.952	0.720	0.509 (0.387 - 0.668)	8.48E-07	*L3MBTL3*
7p14.2	rs6968002	36531229	C/T	0.938	0.958	0.533	0.431 (0.308 - 0.603)	6.32E-07	*AOAH*
8q24.3	rs11785598	144537061	C/T	0.948	0.929	0.328	3.420 (2.223 - 5.266)	6.40E-09	*RHPN1*
9q22.32	rs10512243	97815280	A/G	0.093	0.056	0.913	1.754 (1.411 - 2.181)	2.78E-07	*-*
9q31.2	rs10979327	110160516	G/T	0.112	0.060	0.632	3.851 (2.955 - 5.016)	2.92E-25	*-*
9q33.3	rs1928482	125472269	C/T	0.361	0.303	0.978	1.362 (1.206 - 1.538)	4.87E-07	*DENND1A*
9q34.13	rs1633769	134869098	C/G	0.769	0.803	0.393	0.558 (0.452 - 0.690)	6.07E-08	*-*
9q34.3	rs3811159	136828478	A/G	0.608	0.661	0.683	0.706 (0.616 - 0.810)	6.07E-07	*COL5A1*
10q21.1	rs1903974	53549083	A/G	0.047	0.077	0.865	0.527 (0.409 - 0.680)	4.38E-07	*PRKG1*
11p12	rs7947387	38902742	A/G	0.050	0.069	0.333	0.347 (0.230 - 0.523)	2.45E-07	*-*
12q21.1	rs11178748	70021794	A/G	0.100	0.078	0.897	1.673 (1.366 - 2.046)	5.15E-07	*TSPAN8*
12q23.3	rs7488485	105789959	C/T	0.097	0.073	0.404	2.348 (1.722 - 3.198)	3.17E-08	*RIC8B*
13q21.1	rs9569528	56234110	C/T	0.074	0.051	0.492	2.481 (1.782 - 3.456)	5.27E-08	*-*
14q23.1	rs17097373	59896563	C/T	0.076	0.058	0.770	1.861 (1.448 - 2.391)	9.43E-07	*-*
15q12	rs17651375	25763160	C/T	0.084	0.056	0.934	1.761 (1.417 - 2.189)	2.93E-07	*OCA2*
16q12.1	rs2193094	51123955	G/T	0.557	0.478	0.982	1.337 (1.198 - 1.491)	1.70E-07	*TOX3*
16q21	rs8045513	60720397	A/T	0.059	0.088	0.606	0.430 (0.326 - 0.569)	9.49E-10	*-*
18p11.22	rs17436811	10080771	C/T	0.084	0.058	0.644	2.002 (1.536 - 2.608)	2.20E-07	*-*
19q13.31	rs17725531	49018018	G/T	0.140	0.099	0.930	1.560 (1.311 - 1.858)	5.82E-07	*-*
20p12.2	rs6039942	10376146	A/G	0.100	0.068	0.623	2.005 (1.565 - 2.565)	2.13E-08	*C20orf94*
21q22.11	rs2833424	31774071	A/G	0.293	0.239	0.985	1.365 (1.206 - 1.544)	8.42E-07	*TIAM1*
21q22.11	rs963950	33438577	C/T	0.930	0.883	0.986	1.742 (1.432 - 2.119)	1.24E-08	*IFNAR2*
21q22.13	rs857989	38042001	C/G	0.154	0.111	0.896	1.537 (1.301 - 1.816)	3.72E-07	*KCNJ6*
22q12.3	rs6000351	35340813	A/G	0.093	0.069	0.445	2.100 (1.556 - 2.835)	7.44E-07	*CACNG2*

The table reports, for each SNP, the genomic cytoband, the rs name, the position in build36, the corresponding alleles, the frequency in cases and controls, the imputation quality, the OR and its confidence interval, the pvalue and the most candidate gene within 200 kb. Additional SNPs in the same genomic regions are listed in Additional file 3: Table S2.

One of the identified regions, on chromosome 16q12.1, includes *TOX3*, a gene already associated with breast cancer risk. For this gene, 19 SNPs had *p* < 10^−6^ (Additional file 3: Table S2), supporting a significant susceptibility role for *TOX3* in the Sardinian as well as other populations.

For a second gene already associated with breast cancer, *FGFR2* on chromosome 10q26.13, no SNP had *p* < 10^−6^ but 11 were associated at a less restrictive *p* < 10^−5^ (Additional file 3: Table S2).

No additional SNP with *p* < 1x10^−5^ lay near a gene already associated with breast cancer. Finally, the four SNPs tested in *BRCA1* and those in *BRCA2* all had *p* > 0.05, implying no association with breast cancer in this series. This negative result is consistent with our study’s explicit exclusion of patients with *BRCA* mutations in order to focus on other, low-penetrance loci.

We then attempted to validate the results for ten candidate SNPs, selected among those in genes not already associated with breast cancer, by regenotyping them by TaqMan Assays in a subset of the Sardinian cases and controls (1362 cases and 1514 controls) (Table 3). These markers included nine SNPs in a known gene in the surrounding 200 kb (rs345299, rs6661074, rs13393791, rs17032957, rs1928482, rs1903974, rs11178748, rs963950, rs857989) and one additional SNP (rs10979327) in a desert region but with *p* = 2.92x10^−25^. Of these SNPs, only one, rs345299 reached an association of *p* = 5.38x10^−5^, while it reached *p* = 9.40x10^−7^ in the original analysis (Tables 2 and 3). The difference between the two *p*-values could be partially explained by the call rate, equal to 97 % when using TaqMan. The concordance between genotypes and dosages is consistent with the potential of the signal as a candidate (Table 3). Furthermore, extending the validation to an additional 201 cases and 1467 controls (leading to a total set of 1563 BC cases/2981controls for analyses) the *p*-value reached *p* = 1.16x10^−5^, increasing the robustness of the signal. SNP rs345299 resides in intron 1 of *VAV3*, an oncogene that encodes a guanine nucleotide exchange factor. Both microarray and TaqMan assays suggested that the C allele at this position is associated with disease risk.

**Table 3:** Association results for the 10 candidate SNPs

A						
SNP	corr	FREQ1 cases	FREQ1_CT	N	p-value	n.a. p-value*
rs857989	0.811	0.131	0.106	2866	3.99E-03	4.02E-03
rs11178748	0.824	0.083	0.079	2848	0.289	0.560
rs1903974	0.940	0.043	0.070	2837	5.94E-06	1.34E-05
rs1928482	0.895	0.351	0.313	2771	1.09E-03	2.24E-03
rs10979327	0.697	0.078	0.069	2840	0.178	0.220
rs17032957	0.651	0.749	0.748	2816	0.879	0.910
rs13393791	0.793	0.051	0.041	2847	0.093	0.068
rs6661074	0.806	0.474	0.434	2855	3.62E-03	2.23E-03
rs345299	0.885	0.477	0.530	2789	5.38E-05	9.05E-05
rs17633986	0.809	0.890	0.882	2822	0.553	0.331
**B**						
MARKER	ALLELES	FREQ1_cases	FREQ1_CT	N	n.a. p-value*	
rs857989	C/G	0.130	0.106	4469	8.02E-04	
rs11178748	A/G	0.083	0.088	4432	0.499	
rs1903974	A/G	0.044	0.063	4440	2.40E-04	
rs1928482	C/T	0.350	0.319	4344	3.42E-03	
rs10979327	G/T	0.078	0.074	4456	0.506	
rs17032957	A/G	0.751	0.750	4414	0.865	
rs13393791	C/T	0.050	0.045	4441	0.365	
rs6661074	A/G	0.470	0.442	4416	0.010	
rs345299	A/C	0.481	0.531	4343	1.16E-05	
rs17633986	C/T	0.889	0.877	4451	0.100	

A. Results on Taqman genotypes for 1362 breast cancer cases and 1514 controls included in the GWAS.

B. Results on Taqman genotypes for all 1563 breast cancer cases and 2981 controls.

*n.a. p-value: not adjusted p-value, not corrected for population stratification.

To assess further the significance of this result, we tried to replicate the association of rs345299 with breast cancer risk in a combined analysis of eleven GWAS, comprising 16,195 cases and 18,980 controls, together with data from a custom array (iCOGS) genotyped on 45,290 cases and 41,880 controls of European origin. rs345299 was present on only one of the GWAS arrays but was well imputed in the other GWAS (r^2^ = 0.94 to 0.99) and on the iCOGS array with r^2^ = 0.63. No evidence of association was found in either analyses, nor was there any evidence of association in the iCOGS when analyses were restricted to ER-positive or ER-negative disease (Additional file 3: Table S3). Unfortunately, replication failed suggesting that further analyses are necessary to distinguish whether it is a false positive or an effect specific for the Sardinian population. Testing this hypothesis requires a larger set of patients.

Finally, in a post-hoc analysis, we took advantage of the availability of TNM classification and receptor status data for 921 cases (Table 1) to look for associations between six cancer phenotypes and the cases’ genotypes at 34 candidate SNPs (those in Table 2 plus rs11200014 for *FGFR2*). In all cases, the statistical tests of association gave *p* > 0.05, suggesting that the candidate markers for breast cancer risk are not associated with clinical or molecular subtypes. We also evaluated the association of the 72 breast cancer susceptibility variants identified so far in population meta-analysis in our Sardinian cohort. We were able to detect only rs2981579 *(FGFR2*) and rs3803662 *(TOX3*) with *p-*values of 3.5x10^−6^ and 5.18x10^−6^, respectively (Additional file 3: Table S4). However, if the effect sizes in Sardinian are similar to those reported in other Europeans our sample size is likely underpowered to find associations in the other known genes.

## Discussion

In our Sardinian cohort of breast cancer patients, the predominant molecular subtype was ER+/PR+/HER2- and the percentage of triple-negative cases (7.2 % among those tested) was low. Rates of triple-negative breast cancer have been variably reported in the range of 10 %–20 % in different studies [26]. The somewhat lower rate reported here is in line with findings from two other recent Italian studies: 8.7 % among 2347 patients in Modena [27] and 4.8 % among 2112 patients in Trentino [28].

Our study includes males in the control population, who were recruited for parallel projects. While we have demonstrated that their inclusion in the GWAS does not introduce a bias, we acknowledge this as one of the limitations of the study, along with the low number of cases and controls compared to other reported GWAS in Europeans. Nevertheless, we proceeded with GWAS considering that, in addition to its inter-relatedness, the Sardinian population is relatively stable in towns, with a largely shared lifestyle and diet across the island; thus, both epidemiological and genetic factors are less heterogeneous than in cosmopolitan European populations, with an expected increase in the power to detect associations. Furthermore, GWAS has been successfully done in this population to identify disease-associated alleles in another instance with a limited number of cases/controls [29]. Finally, with our sample size we are completely underpowered to detect low penetrance alleles (OR ~1.05) as those recently described by others. We are instead powered to find alleles with moderate effect size (>1.3) that are poorly tagged in other populations by HapMap SNPs but well captured in Sardinians.

We used SNP genotyping microarrays and identified 33 genomic regions (67 SNPs) associated (at the *p* < 10^−6^ level) with the risk of sporadic, *BRCA*-mutation-negative breast cancer. Of these genomic regions, 20 contained known genes. However, only one of these genes, *TOX3*, has already been associated with breast cancer in other population-based studies. When we lowered the criterion for significance to *p* < 10^−5^, we also identified 11 SNPs in *FGFR2,* another known breast cancer-associated gene.

The attempt to validate 10 of the 67 SNPs selected as candidate markers by singleplex genotyping for technical validation in the original GWA and then in additional samples one, rs345299, that reached a *p-*value of 10^−5^ (*p* = 1.16x10^−5^). The robustness of the signal is supported by the high concordance and by the increase in significance when additional samples are included. This marker, in intron 1 of *VAV3*, was not confirmed to be associated with breast cancer in two other mixed-population cohorts, so that further studies are necessary to clarify whether this marker nevertheless represents a susceptibility locus specific to Sardinians. However, involvement of VAV3 in carcinogenesis is further supported by other recent studies.

VAV3 is a well-characterized guanine nucleotide exchange factor that, upon phosphorylation by receptor tyrosine kinases, participates in signal transduction pathways, resulting in changes in gene expression, cell cycle and cytoskeleton rearrangement [30]. Its activation is thought to be involved in both prostate [31, 32] and breast [33, 34] cancer development and progression, in some cases through stimulation of androgen and ER receptors, respectively [35, 36]. Interestingly, the vast majority (about 75%) of breast cancer patients in our series was positive for ER expression (see Table 1). On the basis of such findings, further studies, such as *VAV3* gene and protein expression in breast cancer samples from Sardinian cases in relation to the genotype, will help in better understanding the association of the SNP with the disease.

Overall, even when the results of analyses in different populations are not mutually confirmatory, they can provide valuable information of wider importance. As an example, *FGFR2* and *TOX3*, which were previously demonstrated to act as risk modifiers in *BRCA1/2* mutation carriers [9, 37], were shown to be associated here in patients without germline mutations in *BRCA1/2*. In this context, *VAV3* may possibly lack a range of gene variations significant for cancer risk in other populations. This would be consistent with the common view that breast cancer patients from different areas may have different genetic backgrounds that influence the impact of low-penetrance susceptibility genes on disease risk.

## Conclusions

In the present study, a case–control GWA study for breast cancer risk was carried out in a large collection of Sardinian breast cancer patients negative for *BRCA1* or *BRCA2* mutations. Among the disease-associated genomic regions, *TOX3* and *FGFR2* genes have been identified as breast cancer susceptibility genes in *BRCA1/2*-wild-type breast cancer patients from Sardinia. Future functional studies on such candidate genes will provide further details about their role in pathogenesis of breast cancer in Sardinian population.

## Availability of supporting data

Genetic results can be downloaded in bulk or searched for SNPs or genes at the web site of the Istituto di Ricerca Genetica e Biomedica (IRGB), National Research Council (CNR), Cagliari, Italy (http://www.irgb.cnr.it/facs/facs.php).

## Additional files

Additional file 1: Figure S1. Quantile-quantile plot obtained with all quality checked SNPs (red dots). The gray area corresponds to the 90 % confidence region from a null distribution of pvalues (generated from 100 simulations).
Additional file 2: Figure S2. A statistical summary of genetic data from Sardinian and HapMap2-CEU samples based on principal component axis one (PCA1) and axis two (PCA2) calculated by using ~40000 independent genome-wide SNPs. Each point represents one individual and is colored by the assigned group (cases, controls and HapMap2-CEU).
Additional file 3: Table S1-S5. **Table S1.** Quality control filtering of genotype data: only the 270742 quality-checked SNPs shared between the two platforms were used in the analysis. **Table S2.** Additional SNP data from the Sardinian breast cancer GWA study. Shown are: (i) additional SNPs associated with breast cancer at the *p*<10^-6^ level but not shown in Table 2, (ii) set of 11 SNPs in *FGFR2* having *p*<10^-5^ and (iii) set of SNPs in *BRCA1* and *BRCA2.***Table S3.** Replication results for the association of rs345299 with breast cancer risk in two larger cohorts, CGEMS and BCAC. **Table S4.** Evaluation of candidate SNPs within known 72 breast cancer susceptibility loci in Sardinia cohort (1,367 cases and 1,658 controls). **Table S5.** Gender-based allele frequency for the most significant SNPs reported in Table 2.
Additional file 4: Table S6. Most significant SNPs in 33 genomic regions associated with sporadic, BRCA-mutation-negative breast cancer patients in the Sardinian population, excluding the nine male breast cancer cases.
