# Supplementary figures and images for: Genome-wide association study of susceptibility loci for breast cancer in Sardinian population
Source: BMC Cancer. 2015 May 10;15:383. doi: 10.1186/s12885-015-1392-9 (PMC4434540; doi:10.1186/s12885-015-1392-9)

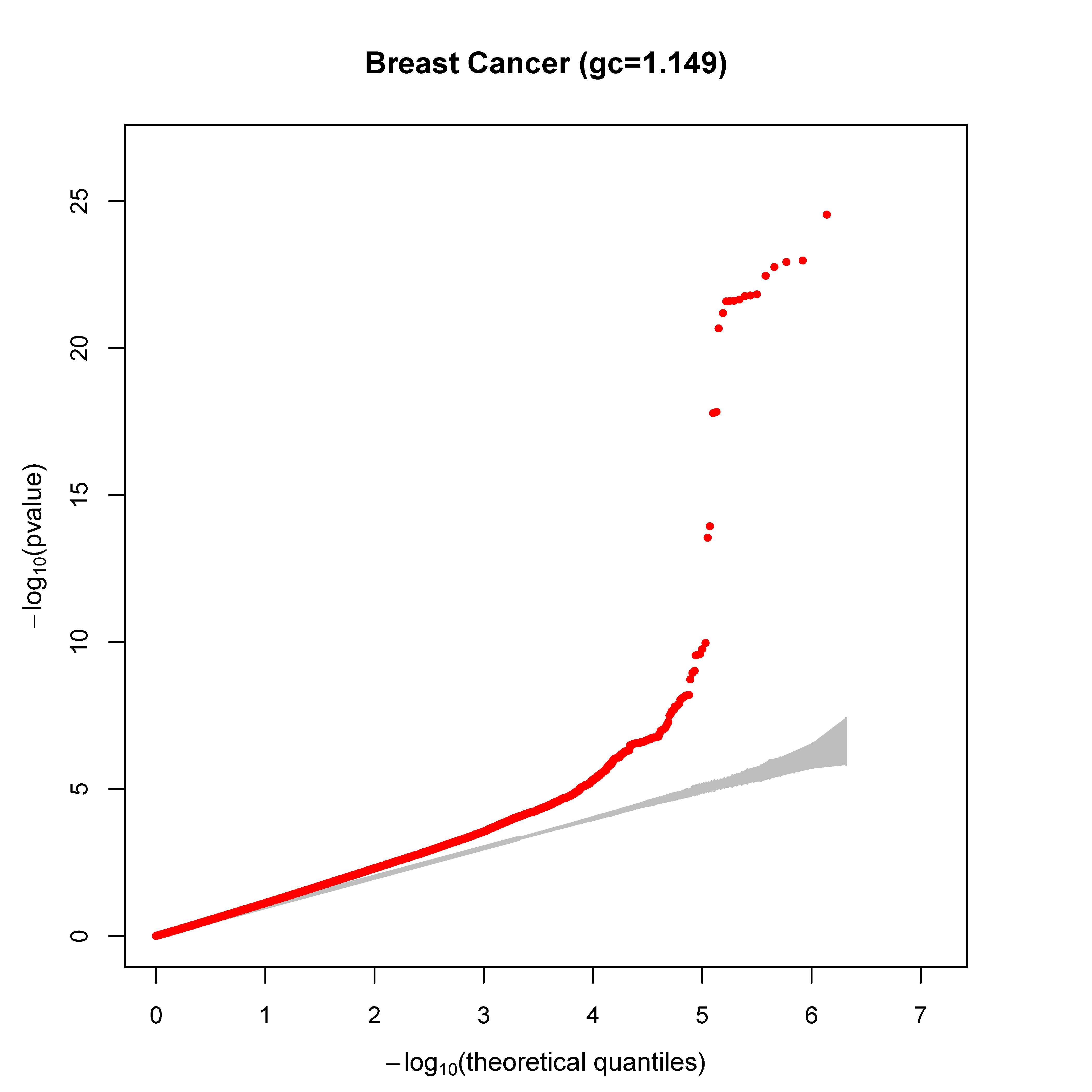

Supplement: Additional file 1: Figure S1. — Quantile-quantile plot obtained with all quality checked SNPs (red dots). The gray area corresponds to the 90 % confidence region from a null distribution of pvalues (generated from 100 simulations). [file 12885_2015_1392_MOESM1_ESM.tiff]

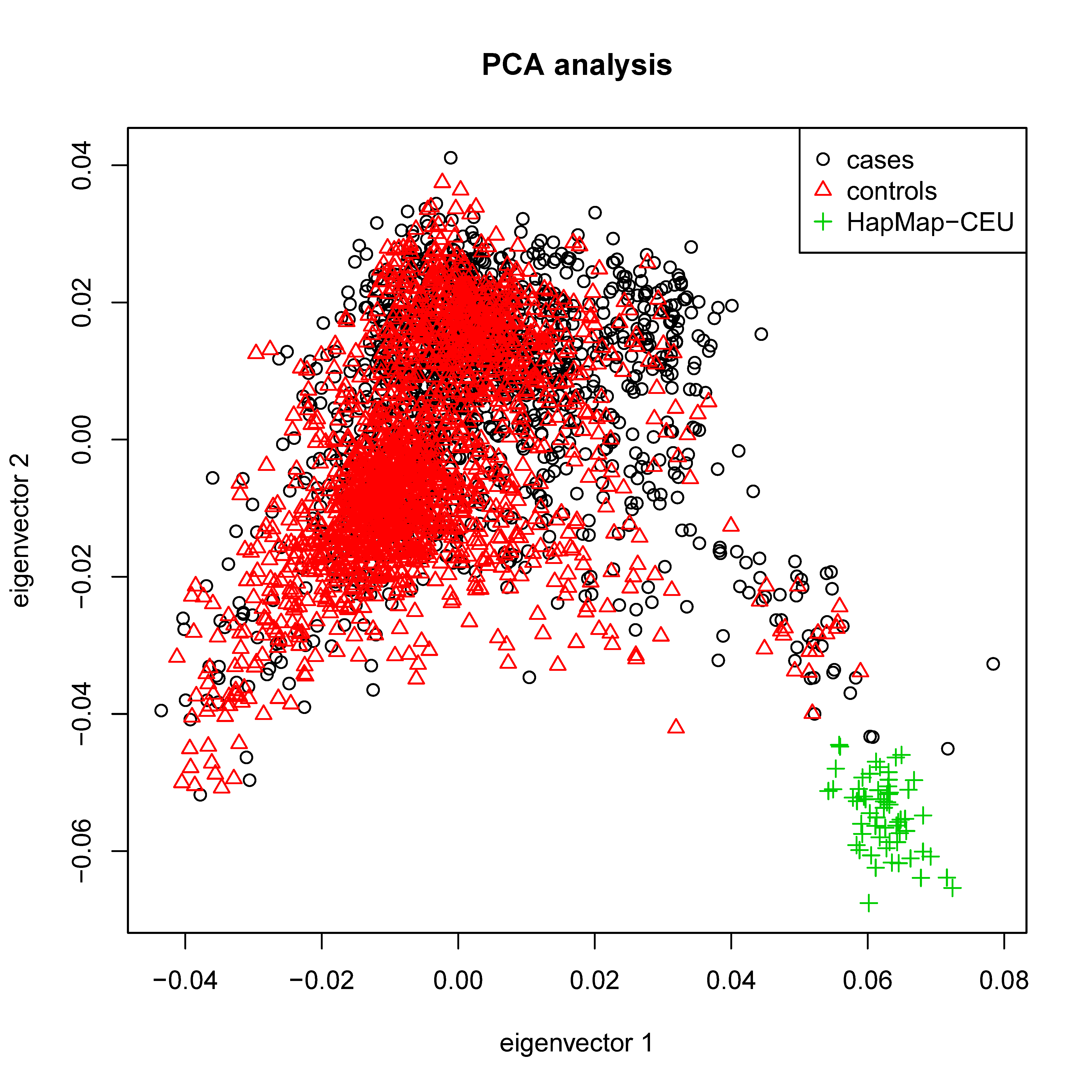

Supplement: Additional file 2: Figure S2. — A statistical summary of genetic data from Sardinian and HapMap2-CEU samples based on principal component axis one (PCA1) and axis two (PCA2) calculated by using ~40000 independent genome-wide SNPs. Each point represents one individual and is colored by the assigned group (cases, controls and HapMap2-CEU). [file 12885_2015_1392_MOESM2_ESM.tiff]
