# Supplementary material for: Genome-wide association study of susceptibility loci for breast cancer in Sardinian population
Source: BMC Cancer. 2015 May 10;15:383. doi: 10.1186/s12885-015-1392-9 (PMC4434540; doi:10.1186/s12885-015-1392-9)
Supplement: Additional file 3: Table S1-S5. — Table S1. Quality control filtering of genotype data: only the 270742 quality-checked SNPs shared between the two platforms were used in the analysis. Table S2. Additional SNP data from the Sardinian breast cancer GWA study. Shown are: (i) additional SNPs associated with breast cancer at the p<10-6 level but not shown in Table 2, (ii) set of 11 SNPs in FGFR2 having p<10-5 and (iii) set of SNPs in BRCA1 and BRCA2.Table S3. Replication results for the association of rs345299 with breast cancer risk in two larger cohorts, CGEMS and BCAC. Table S4. Evaluation of candidate SNPs within known 72 breast cancer susceptibility loci in Sardinia cohort (1,367 cases and 1,658 controls). Table S5. Gender-based allele frequency for the most significant SNPs reported in Table 2. [file 12885_2015_1392_MOESM3_ESM.docx]

**Additional file 3**

**Table S1.** Quality control filtering of genotype data: only the 270742 quality-checked SNPs shared between the two platforms were used in the analysis

|  | | 500K Array Set | SNP Array 6.0 | Shared SNPs |
| --- | --- | --- | --- | --- |
| SNPs eliminated, n | |  |  |  |
|  | Sample call rate <95% | 41304 | 237861 | 833 |
|  | Minor AF <5% | 119565 | 244953 | 655 |
|  | Harvey-Weinberg equilibrium p<10^-6^ | 7704 | 90311 | 269 |
|  | AF difference >10% between arrays | NA | NA | 14190 |
| SNPs not eliminated, n | | 341229 | 492570 | 270742 |

AF, allele frequency; NA, not applicable

**Table S2.** Additional SNP data from the Sardinian breast cancer GWA study. Shown are: (i) additional SNPs associated with breast cancer at the *p*<10^-6^ level but not shown in Table 2, (ii) set of 11 SNPs in *FGFR2* having *p*<10^-5^ and (iii) set of SNPs in *BRCA1* and *BRCA2*

| **Chromosomal region** | **SNP** | **Position** | **Alleles** | **RSQR** | **Frequency** | | **OR (CI 95%)** | **P** | **Gene** |
| --- | --- | --- | --- | --- | --- | --- | --- | --- | --- |
|  |  |  |  |  | **Cases** | **Controls** |  |  |  |
| **Additional SNPs significant at p<10-6** | | | | | | | | | |
| 2p25.3 | rs10192670 | 3235518 | C/G | 0.958 | 0.075 | 0.04 | 1.848 (1.455-2.347) | 3.17E-07 | *TSSC1* |
| 2p25.3 | rs10198129 | 3241821 | C/T | 0.992 | 0.074 | 0.039 | 1.860 (1.466-2.356) | 2.03E-07 | *TSSC1* |
| 6q23.1 | rs12526192 | 130498364 | C/T | 0.719 | 0.074 | 0.049 | 1.966 (1.497-2.582) | 8.51E-07 | *L3MBTL3* |
| 6q23.1 | rs2068957 | 130502285 | A/G | 0.694 | 0.074 | 0.049 | 1.986 (1.506-2.618) | 8.70E-07 | *L3MBTL3* |
| 6q23.1 | rs7775285 | 130494293 | A/C | 0.72 | 0.074 | 0.048 | 1.965 (1.497-2.582) | 8.51E-07 | *L3MBTL3* |
| 6q23.1 | rs987836 | 130502324 | A/G | 0.684 | 0.926 | 0.951 | 0.502 (0.380-0.663) | 8.85E-07 | *L3MBTL3* |
| 9q33.3 | rs10986088 | 125483780 | C/T | 0.912 | 0.656 | 0.711 | 0.717 (0.633-0.813) | 2.06E-07 | *DENND1A* |
| 9q33.3 | rs7857068 | 125475640 | A/T | 0.97 | 0.64 | 0.696 | 0.737 (0.652-0.832) | 6.95E-07 | *DENND1A* |
| 9q33.3 | rs879070 | 125473035 | C/T | 0.977 | 0.361 | 0.303 | 1.361 (1.205-1.537) | 5.10E-07 | *DENND1A* |
| 9q33.3 | rs913783 | 125473924 | A/G | 0.971 | 0.64 | 0.696 | 0.736 (0.652-0.832) | 6.53E-07 | *DENND1A* |
| 10q21.1 | rs12260890 | 53559415 | C/T | 0.963 | 0.04 | 0.071 | 0.530 (0.411-0.684) | 4.97E-07 | *PRKG1* |
| 10q21.1 | rs16927026 | 53557035 | A/G | 0.964 | 0.96 | 0.929 | 1.887 (1.463-2.435) | 4.93E-07 | *PRKG1* |
| 10q21.1 | rs3862583 | 53540581 | A/G | 0.967 | 0.042 | 0.072 | 0.539 (0.421-0.692) | 6.54E-07 | *PRKG1* |
| 10q21.1 | rs4935029 | 53530494 | C/T | 0.983 | 0.042 | 0.073 | 0.550 (0.431-0.703) | 9.14E-07 | *PRKG1* |
| 16q12.1 | rs1075367 | 51112503 | A/G | 0.927 | 0.549 | 0.473 | 1.342 (1.200-1.500) | 2.45E-07 | *TOX3* |
| 16q12.1 | rs1123428 | 51135336 | A/T | 0.996 | 0.558 | 0.478 | 1.333 (1.196-1.484) | 1.80E-07 | *TOX3* |
| 16q12.1 | rs11642645 | 51116408 | A/C | 0.93 | 0.55 | 0.473 | 1.343 (1.201-1.502) | 2.28E-07 | *TOX3* |
| 16q12.1 | rs12443621 | 51105538 | A/G | 0.925 | 0.451 | 0.527 | 0.745 (0.666-0.833) | 2.56E-07 | *TOX3* |
| 16q12.1 | rs12598982 | 51094386 | C/T | 0.92 | 0.451 | 0.527 | 0.746 (0.666-0.833) | 2.84E-07 | *TOX3* |
| 16q12.1 | rs1362546 | 51107436 | C/T | 0.926 | 0.451 | 0.527 | 0.745 (0.666-0.833) | 2.48E-07 | *TOX3* |
| 16q12.1 | rs1420529 | 51116174 | G/T | 0.927 | 0.549 | 0.473 | 1.342 (1.200-1.500) | 2.41E-07 | *TOX3* |
| 16q12.1 | rs1420533 | 51121127 | A/G | 0.971 | 0.557 | 0.478 | 1.339 (1.200-1.494) | 1.74E-07 | *TOX3* |
| 16q12.1 | rs2193094 | 51123955 | G/T | 0.982 | 0.557 | 0.478 | 1.337 (1.198-1.491) | 1.70E-07 | *TOX3* |
| 16q12.1 | rs3112580 | 51129106 | C/T | 0.996 | 0.558 | 0.478 | 1.333 (1.196-1.484) | 1.78E-07 | *TOX3* |
| 16q12.1 | rs3112581 | 51129034 | A/G | 0.996 | 0.442 | 0.522 | 0.750 (0.673-0.835) | 1.75E-07 | *TOX3* |
| 16q12.1 | rs4783780 | 51128937 | A/C | 0.995 | 0.442 | 0.522 | 0.750 (0.673-0.835) | 1.71E-07 | *TOX3* |
| 16q12.1 | rs4784222 | 51095087 | C/G | 0.922 | 0.549 | 0.473 | 1.341 (1.199-1.499) | 2.79E-07 | *TOX3* |
| 16q12.1 | rs7190749 | 51101579 | A/G | 0.923 | 0.451 | 0.528 | 0.745 (0.666-0.833) | 2.69E-07 | *TOX3* |
| 16q12.1 | rs8046979 | 51113243 | A/G | 0.908 | 0.457 | 0.533 | 0.741 (0.662-0.831) | 2.06E-07 | *TOX3* |
| 16q12.1 | rs9302556 | 51101146 | A/C | 0.922 | 0.549 | 0.473 | 1.341 (1.200-1.500) | 2.75E-07 | *TOX3* |
| 16q12.1 | rs9931232 | 51130333 | A/G | 0.997 | 0.558 | 0.478 | 1.333 (1.196-1.484) | 1.80E-07 | *TOX3* |
| 16q12.1 | rs9933556 | 51105803 | C/T | 0.926 | 0.451 | 0.527 | 0.745 (0.666-0.833) | 2.49E-07 | *TOX3* |
| 16q12.1 | rs9933638 | 51097098 | A/G | 0.922 | 0.451 | 0.528 | 0.746 (0.666-0.833) | 2.77E-07 | *TOX3* |
| 21q22.13 | rs702864 | 38040137 | A/G | 0.799 | 0.17 | 0.128 | 1.527 (1.290-1.807) | 7.69E-07 | *KCNJ6* |
| **SNPs in *FGFR2* significant at p<10-5** | | | | | | | | | |
| 10q26.13 | rs1078806 | 123328965 | A/G | 0.966 | 0.431 | 0.494 | 0.769 (0.688-0.860) | 3.53E-06 | *FGFR2* |
| 10q26.13 | rs11200014 | 123324920 | A/G | 0.952 | 0.568 | 0.506 | 1.303 (1.164-1.456) | 3.51E-06 | *FGFR2* |
| 10q26.13 | rs1219648 | 123336180 | A/G | 0.883 | 0.446 | 0.505 | 0.766 (0.683-0.860) | 6.70E-06 | *FGFR2* |
| 10q26.13 | rs2420946 | 123341314 | C/T | 0.873 | 0.447 | 0.506 | 0.765 (0.681-0.861) | 7.12E-06 | *FGFR2* |
| 10q26.13 | rs2860197 | 123341292 | A/G | 0.874 | 0.447 | 0.506 | 0.765 (0.681-0.861) | 7.05E-06 | *FGFR2* |
| 10q26.13 | rs2912774 | 123338652 | G/T | 0.878 | 0.447 | 0.505 | 0.766 (0.681-0.861) | 6.90E-06 | *FGFR2* |
| 10q26.13 | rs2912780 | 123327107 | C/T | 0.956 | 0.569 | 0.506 | 1.302 (1.164-1.456) | 3.51E-06 | *FGFR2* |
| 10q26.13 | rs2936870 | 123338892 | C/T | 0.877 | 0.447 | 0.505 | 0.766 (0.681-0.861) | 6.92E-06 | *FGFR2* |
| 10q26.13 | rs2981578 | 123330301 | C/T | 0.843 | 0.604 | 0.542 | 1.323 (1.174-1.491) | 4.48E-06 | *FGFR2* |
| 10q26.13 | rs2981579 | 123327325 | A/G | 0.963 | 0.569 | 0.506 | 1.301 (1.163-1.455) | 3.54E-06 | *FGFR2* |
| 10q26.13 | rs2981582 | 123342307 | A/G | 0.872 | 0.553 | 0.494 | 1.306 (1.161-1.469) | 7.18E-06 | *FGFR2* |
| **Non-significant SNPs in *BRCA1/2*** | | | | | | | | | |
| 13q13.1 | rs206079 | 31818618 | A/G | 0.986 | 0.534 | 0.533 | 1.012 (0.907-1.129) | 0.836 | *BRCA2* |
| 13q13.1 | rs9567578 | 31819944 | A/G | 0.997 | 0.777 | 0.783 | 1.010 (0.886-1.152) | 0.878 | *BRCA2* |
| 13q13.1 | rs206081 | 31820136 | C/T | 0.996 | 0.759 | 0.767 | 0.949 (0.837-1.076) | 0.418 | *BRCA2* |
| 13q13.1 | rs11571725 | 31836881 | A/G | 0.997 | 0.817 | 0.826 | 1.011 (0.876-1.167) | 0.885 | *BRCA2* |
| 17q21.31 | rs8176318 | 38450800 | A/C | 0.991 | 0.306 | 0.327 | 0.924 (0.822-1.036) | 0.177 | *BRCA1* |
| 17q21.31 | rs8176198 | 38484063 | A/T | 0.993 | 0.691 | 0.669 | 1.092 (0.973-1.226) | 0.135 | *BRCA1* |
| 17q21.31 | rs4793197 | 38485428 | A/G | 0.998 | 0.305 | 0.326 | 0.919 (0.819-1.032) | 0.152 | *BRCA1* |
| 17q21.31 | rs8176092 | 38523755 | G/T | 0.755 | 0.235 | 0.256 | 0.881 (0.764-1.017) | 0.084 | *BRCA1* |

**Table S3.** Replication results for the association of rs345299 with breast cancer risk in two larger cohorts, CGEMS and BCAC.

| **Study** | **Controls** | **Cases** | **Allele frequency** | **Imputation r^2^** | **OR (95%CI)** | **P-value** |
| --- | --- | --- | --- | --- | --- | --- |
| *CGEMS cohort* |  |  |  |  |  |  |
| CGEMS Breast | 1140 | 1143 |  |  | 0.98 (0.87-1.10) | 0.80 |
| CGEMS Breast + NHS2 cases | 1140 | 1188 |  |  | 0.97 (0.86-1.09) | 0.65 |
| Poland breast plus controls | 583 | 543 |  |  | 0.89 (0.75-1.05) | 0.16 |
| Single analysis of everything above | 1723 | 1731 |  |  | 0.94 (0.85-1.03) | 0.24 |
|  |  |  |  |  |  |  |
| *BCAC cohorts* |  |  |  |  |  |  |
| ABCFS | 285 | 282 | 0.43 | 0.98 | 1.18 (0.93-1.49) | 0.17 |
| DFBBCS | 3255 | 464 | 0.43 | 0.98 | 0.97 (0.85-1.12) | 0.68 |
| BBCS | 5190 | 1609 | 0.42 | 0.98 | 1.03 (0.93-1.15) | 0.57 |
| GC-HBOC | 477 | 634 | 0.42 | Genotyped | 1.06 (0.90-1.26) | 0.49 |
| UK2 | 5190 | 3628 | 0.44 | 0.98 | 0.97 (0.91-1.05) | 0.48 |
| HEBCS | 1012 | 810 | 0.42 | 0.99 | 0.99 (0.86-1.14) | 0.89 |
| MARIE | 470 | 708 | 0.44 | 0.98 | 0.93 (0.78-1.10) | 0.40 |
| SASBAC | 756 | 790 | 0.40 | 0.98 | 1.02 (0.89-1.18) | 0.76 |
| BPC3 | 1998 | 2305 | 0.43 |  | 0.99 (0.90-1.08) | 0.78 |
| BCFR | 2457 | 3486 | 0.45 | 0.94 | 0.94 (0.87-1.02) | 0.13 |
| TNBCC | 3399 | 1562 | 0.42 | 0.92-0.94 | 0.98 (0.88-1.08) | 0.64 |
| Combined GWAS | 18,980 | 16,195 | 0.43 |  | 0.99 (0.96-1.03) | 0.63 |
| iCOGS (41 studies) | 43,612 | 48,155 | 0.41 | 0.63 | 1.00 (0.97-1.02) | 0.48 |
| Combined GWAS+iCOGS |  |  | 0.41 |  | 0.99 (0.97-1.01) | 0.57 |
| iCOGS ER-positive | 42,111 | 27,078 |  |  | 1.01 (0.98-1.04) | 0.28 |
| iCOGS ER-negative | 42,468 | 7333 |  |  | 0.99 (0.96-1.06) | 0.24 |

**Table S4.** Evaluation of candidate SNPs within known 72 breast cancer susceptibility loci in Sardinia cohort (1,367 cases and 1,658 controls)

| **Lead SNP** | **Chr.** | **Position** | **Alleles** | **Combined GWAS [OR (95%CI)]** | **Frequency**  **cases** | **controls** | **RSQR** | **OR (95%CI)** | **p-value** | **Het p-value** | **power (10^-6^)** | **power (10^-8^)** | **Locus** |
| --- | --- | --- | --- | --- | --- | --- | --- | --- | --- | --- | --- | --- | --- |
| rs616488 | 1 | 10488802 | G/A | 0.94 (0.92–0.96) | 0.3077 | 0.3105 | 0.8148 | 1.03 (0.91-1.18) | 0.6301 | 0.1665 | 36168 | 49002 | *PEX14* |
| rs6678914 | 1 | 20045399 | G/A | 1.10 (1.06–1.13) | 0.5945 | 0.5894 | 0.9962 | 1.06 (0.95-1.18) | 0.3357 | 0.4737 | 13230 | 17925 | *LGR6-PTPN22-BCL2* |
| rs11552449 | 1 | 114249912 | T/C | 1.07 (1.04–1.09) | 0.8309 | 0.8182 | 0.7913 | 1.12 (0.95-1.31) | 0.1681 | 0.5866 | 44975 | 60909 | *L15-AP4B1-DCLRE1B-HIPK1* |
| rs11249433 | 1 | 120982136 | G/A | 1.14 (1.10-1.19) | 0.4581 | 0.4525 | 0.4745 | 1.01 (0.86-1.18) | 0.9211 | 0.1426 | 6849 | 9280 | *1p11.2* |
| rs4245739 | 1 | 202785465 | C/A | 1.14 (1.10–1.18) | 0.3374 | 0.3578 | 0.9784 | 0.93 (0.83-1.04) | 0.2162 | 0.0009 | 7380 | 9998 | *MDM4* |
| rs12710696 | 2 | 19184284 | T/C | 1.10 (1.06–1.13 | 0.3606 | 0.3594 | 0.9978 | 1.02 (0.91-1.14) | 0.7203 | 0.205 | 13813 | 18715 | *2p24.1* |
| rs4849887 | 2 | 120961592 | T/C | 0.91 (0.88–0.94) | 0.1218 | 0.1287 | 0.9812 | 0.89 (0.75-1.05) | 0.1593 | 0.7756 | 30966 | 41954 | *2q14.2* |
| rs2016394 | 2 | 172681217 | A/G | 0.95 (0.93–0.97) | 0.4657 | 0.4699 | 0.4594 | 0.98 (0.84-1.15) | 0.7959 | 0.7074 | 44712 | 60578 | *METAP1D-DLX1-DLX2* |
| rs1550623 | 2 | 173921140 | G/A | 0.94 (0.92–0.97) | 0.1209 | 0.117 | 0.8638 | 1.10 (0.92-1.33) | 0.2971 | 0.0936 | 74658 | 101149 | *CDCA7* |
| rs1045485 | 2 | 201857834 | C/G | 0.90 (0.85-0.96) | 0.0777 | 0.0836 | 0.9862 | 0.84 (0.68-1.03) | 0.0923 | 0.5209 | 36912 | 50009 | *CASP8* |
| rs13387042 | 2 | 217614077 | G/A | 0.83 (0.80-0.86) | 0.4334 | 0.4572 | 0.9897 | 0.91 (0.81-1.01) | 0.0786 | 0.1229 | 3461 | 4689 | *2q35* |
| rs16857609 | 2 | 218004753 | T/C | 1.08 (1.06–1.10) | 0.2689 | 0.2619 | 0.974 | 1.02 (0.90-1.15) | 0.7835 | 0.3465 | 24921 | 33765 | *DIRC3* |
| rs6762644 | 3 | 4717276 | G/A | 1.07 (1.04–1.09) | 0.1986 | 0.1852 | 0.9959 | 1.07 (0.93-1.23) | 0.3353 | 0.9961 | 40486 | 54852 | *ITPR1-EGOT* |
| rs4973768 | 3 | 27391017 | T/C | 1.14 (1.09-1.18) | 0.5469 | 0.521 | 0.9543 | 1.11 (1.00-1.24) | 0.0608 | 0.6575 | 6889 | 9334 | *SLC4A7* |
| rs12493607 | 3 | 30657943 | C/G | 1.06 (1.03–1.08) | 0.3154 | 0.3038 | 0.9653 | 1.08 (0.95-1.21) | 0.2286 | 0.8114 | 39917 | 54081 | *TGFBR2* |
| rs9790517 | 4 | 106304227 | T/C | 1.05 (1.03–1.08) | 0.2908 | 0,2753 | 0.8643 | 1.19 (1.04-1.36) | 0.0101 | 0.0693 | 60018 | 81315 | *TET2* |
| rs6828523 | 4 | 176083001 | A/C | 0.90 (0.87–0.92) | 0.0768 | 0.0851 | 0.9983 | 0.87 (0.71-1.06) | 0.1624 | 0.721 | 36745 | 49783 | *ADAM29* |
| rs10069690 | 5 | 1332790 | T/C | 1.04 (0.98-1.11) | 0.271 | 0.2711 | 0.1204 | 0.99 (0.70-1.40) | 0.9462 | 0.7784 | 95387 | 129234 | *TERT* |
| rs10941679 | 5 | 44742255 | G/A | 1.17 (1.12-1.23) | 0.2359 | 0.21 | 0.6575 | 1.20 (1.02-1.41) | 0.0258 | 0.7621 | 6615 | 8963 | *5p12* |
| rs889312 | 5 | 56067641 | C/A | 1.16 (1.11-1.21) | 0.3494 | 0.311 | 0.9746 | 1.21 (1.08-1.36) | 0,0013 | 0.5182 | 5894 | 7986 | *MAP3K1* |
| rs10472076 | 5 | 58219818 | C/T | 1.05 (1.03–1.07) | 0.3642 | 0.3556 | 0.7575 | 1.04 (0.92-1.19) | 0.5265 | 0.9189 | 53002 | 71809 | *RAB3C* |
| rs1353747 | 5 | 58373238 | G/T | 0.92 (0.89–0.95) | 0.0596 | 0.0753 | 0.8424 | 0.76 (0.60-0.96) | 0.0201 | 0.1091 | 68483 | 92783 | *PDE4D* |
| rs1432679 | 5 | 158176661 | C/T | 1.07 (1.05–1.09) | 0.5803 | 0.5532 | 0.9548 | 1.10 (0.98-1.23) | 0.0930 | 0.6238 | 26118 | 35386 | *EBF1* |
| rs11242675 | 6 | 1263878 | C/T | 0.94 (0.92–0.96) | 0.3005 | 0.2951 | 0.9653 | 1.05 (0.93-1.18) | 0.4114 | 0.0708 | 36989 | 50115 | *FOXQ1* |
| rs204247 | 6 | 13830502 | G/A | 1.05 (1.03–1.07) | 0.4073 | 0.4057 | 0.9951 | 1.00 (0.90-1.12) | 0.9509 | 0.4203 | 50700 | 68691 | *RANBP9* |
| rs17530068 | 6 | 82249828 | G/A | 1.09 (1.04-1.14) | 0.3375 | 0.3267 | 0.8936 | 1.06 (0.94-1.20) | 0.3497 | 0.6698 | 17536 | 23759 | *6q14.1* |
| rs3757318 | 6 | 151955806 | A/G | 1.21 (1.13-1.31) | 0.1329 | 0.1402 | 0.8378 | 1.01 (0.85-1.19) | 0.9408 | 0.0543 | 6398 | 8669 | *ESR1* |
| rs2046210 | 6 | 151990059 | A/G | 1.11 (1.07-1.16) | 0.3804 | 0.3569 | 0.9949 | 1.15 (1.03-1.29) | 0.0128 | 0.539 | 11417 | 15469 | *ESR1* |
| rs720475 | 7 | 143705862 | A/G | 0.94 (0.92–0.96) | 0.298 | 0.2888 | 0.9928 | 1.04 (0.93-1.17) | 0.4778 | 0.0853 | 37336 | 50584 | *ARHGEF5-NOBOX* |
| rs9693444 | 8 | 29565535 | A/C | 1.07 (1.05–1.09) | 0.3878 | 0.351 | 0.9981 | 1.09 (0.97-1.21) | 0.151 | 0.804 | 27256 | 36927 | *8p21.1* |
| rs6472903 | 8 | 76392856 | G/T | 0.91 (0.89–0.93) | 0.1391 | 0.1553 | 0.6383 | 0.86 (0.71-1.05) | 0.131 | 0.5862 | 26919 | 36471 | *8q21.11* |
| rs2943559 | 8 | 76580492 | G/A | 1.13 (1.09–1.17) | 0.0858 | 0.0871 | 0.9889 | 0.97 (0.80-1.17) | 0.7201 | 0.1144 | 4587 | 6215 | *HNF4G* |
| rs13281615 | 8 | 128424800 | G/A | 1.10 (1.14-1.19) | 0.5179 | 0.4793 | 0.9674 | 1.15 (1.03-1.28) | 0.0146 | 0.5355 | 12889 | 17463 | *8q24* |
| rs11780156 | 8 | 129263823 | T/C | 1.07 (1.04–1.10) | 0.1725 | 0.1713 | 0.9769 | 0.98 (0.85-1.14) | 0.805 | 0.2555 | 43963 | 59562 | *MIR1208* |
| rs1011970 | 9 | 22052134 | T/G | 1.10 (1.05-1.16) | 0.144 | 0.1408 | 0.5589 | 0.96 (0.79-1.18) | 0.7207 | 0.2183 | 25562 | 34632 | *CDKN2A/B* |
| rs10759243 | 9 | 109345936 | A/C | 1.06 (1.03–1.08) | 0.3207 | 0.3068 | 0.9559 | 1.07 (0.95-1.21) | 0,2335 | 0,8212 | 39639 | 53704 | *9q31.2* |
| rs865686 | 9 | 109928299 | G/T | 0.89 (0.86-0.93) | 0.4259 | 0.4673 | 0.9285 | 0.86 (0.77-0.96) | 0.0076 | 0.5516 | 8773 | 11886 | *9q31* |
| rs2380205 | 10 | 5926740 | T/C | 0.94 (0.90-0.97) | 0.5772 | 0.6059 | 0.9628 | 0.95 (0.85-1.06) | 0.394 | 0.8279 | 31480 | 42650 | *ANKRD16* |
| rs7072776 | 10 | 22072948 | A/G | 1.07 (1.05–1.09) | 0.3187 | 0.2923 | 0.7941 | 1.14 (1.00-1.29) | 0.0561 | 0.3734 | 29812 | 40390 | *MLLT10-DNAJC1* |
| rs11814448 | 10 | 22355849 | C/A | 1.26 (1.18–1.35) | 0.0307 | 0.0248 | 0.9359 | 1.20 (0.85-1.70) | 0.2941 | 0.8026 | 18416 | 24950 | *DNAJC1* |
| rs10995190 | 10 | 63948688 | A/G | 0.84 (0.80-0.89) | 0.1323 | 0.1225 | 0.8898 | 1.18 (1.00-1.40) | 0,0552 | 0.0001 | 9255 | 12539 | *ZNF365* |
| rs704010 | 10 | 80511154 | T/C | 1.12 (1.08-1.17) | 0.4536 | 0.4265 | 0.9995 | 1.12 (1.00-1.25) | 0.0411 | 0.9911 | 9203 | 12469 | *ZMIZ1* |
| rs7904519 | 10 | 114763917 | G/A | 1.06 (1.04–1.08) | 0.5672 | 0.554 | 0.9864 | 1.05 (0.94-1.17) | 0.4103 | 0.8454 | 35077 | 47523 | *TCF7L2* |
| rs11199914 | 10 | 123083891 | T/C | 0.95 (0.93–0.97) | 0.2724 | 0.2758 | 0.2825 | 0.88 (0.70-1.11) | 0.2921 | 0.5416 | 56462 | 76498 | *10q26.12* |
| rs2981579 | 10 | 123327325 | A/G | 1.35 (1.29-1.40) | 0.5689 | 0.5061 | 0.9625 | 1.30 (1.16-1.45) | 3,543E-06 | 0.5359 | 1339 | 1814 | *FGFR2* |
| rs3817198 | 11 | 1865582 | C/T | 1.06 (1.01-1.10) | 0.3372 | 0.3044 | 0.9878 | 1.12 (1.00-1.26) | 0.0576 | 0.3843 | 39243 | 53168 | *LSP1* |
| rs3903072 | 11 | 65339642 | T/G | 0.95 (0.93–0.96) | 0.4871 | 0.5093 | 0.9944 | 0.95 (0.85-1.05) | 0.3131 | 0.9321 | 44462 | 60239 | *DKFZp761E198-OVOL1-SNX32-CFL1-MUS81* |
| rs614367 | 11 | 69037945 | T/C | 1.31 (1.23-1.38) | 0,0997 | 0.0921 | 0.9891 | 1.10 (0.92-1.33) | 0.2945 | 0.0799 | 4208 | 5701 | *11q13* |
| rs11820646 | 11 | 128966381 | T/C | 0.95 (0.93–0.97) | 0.3541 | 0.3835 | 0.9912 | 0.91 (0.81-1.02) | 0.107 | 0.466 | 47974 | 64997 | *11q24.3* |
| rs12422552 | 12 | 14305198 | C/G | 1.05 (1.03–1.07) | 0.2737 | 0.2659 | 0.5006 | 1.02 (0.86-1.21) | 0.8484 | 0.7195 | 61744 | 83653 | *12p13.1* |
| rs10771399 | 12 | 28046347 | G/A | 0.82 (0.77-0.87) | 0.0561 | 0.0652 | 0.899 | 0.77 (0.61-0.98) | 0.0351 | 0.647 | 14080 | 19076 | *PTHLH* |
| rs17356907 | 12 | 94551890 | G/A | 0.91 (0.89–0.93) | 0.2753 | 0.2843 | 0.1799 | 0.73 (0.55-0.97) | 0.0295 | 0.1266 | 16640 | 22544 | *NTN4* |
| rs1292011 | 12 | 114320905 | G/A | 0.91 (0.87-0.94) | 0.3734 | 0.3905 | 0.6387 | 0.95 (0.83-1.09) | 0.4953 | 0.5343 | 14079 | 19075 | *12q24* |
| rs11571833 | 13 | 31870626 | T/A | 1.26 (1.14–1.39) | 0.0121 | 0.0092 | 0.4665 | 1.85 (0.86-3.95) | 0.1126 | 0.3302 | 47361 | 64166 | *BRCA2-N4BP2L1-N4BP2L2* |
| rs2236007 | 14 | 36202520 | A/G | 0.93 (0.91–0.95) | 0.2042 | 0.2146 | 0,7113 | 0.90 (0.77-1.05) | 0.1767 | 0.6698 | 34164 | 46287 | *PAX9-SLC25A21* |
| rs2588809 | 14 | 67730181 | T/C | 1.08 (1.05–1.11) | 0.2388 | 0.239 | 0,9346 | 1.02 (0.89-1.16) | 0.8102 | 0.3731 | 26650 | 36107 | *RAD51L1* |
| rs999737 | 14 | 68104435 | T/C | 0.87 (0.83-0.91) | 0.2128 | 0.2286 | 0,96 | 0.92 (0.80-1.05) | 0.2049 | 0.458 | 9091 | 12316 | *RAD51L1* |
| rs941764 | 14 | 90910822 | G/A | 1.06 (1.04–1.09) | 0.3126 | 0.3213 | 0,9902 | 0.99 (0.89-1.12) | 0.9153 | 0.2896 | 39358 | 53323 | *CCDC88C* |
| rs3803662 | 16 | 51143842 | A/G | 1.28 (1.23-1.34) | 0.3857 | 0.3311 | 0,9388 | 1.31 (1.16-1.47) | 5,185E-06 | 0.751 | 2045 | 2771 | *TOX3* |
| rs17817449 | 16 | 52370868 | G/T | 0.93 (0.91–0.95) | 0.4635 | 0.4785 | 0,987 | 0.99 (0.89-1.10) | 0.836 | 0.2721 | 22337 | 30263 | *MIR1972-2-FTO* |
| rs11075995 | 16 | 52412792 | T/A | 1.11 (1.07–1.15) | 0.775 | 0.7642 | 0,5421 | 1.05 (0.88-1.25) | 0.5663 | 0,5568 | 14738 | 19967 | *KIAA1752-FTO* |
| rs13329835 | 16 | 79208306 | G/A | 1.08 (1.05–1.10) | 0.2995 | 0.278 | 0.9932 | 1.06 (0.94-1.19) | 0.329 | 0.7674 | 23732 | 32154 | *CDYL2* |
| rs6504950 | 17 | 50411470 | A/G | 0.91 (0.87-0.95) | 0.2452 | 0.2589 | 0.9856 | 0.97 (0.86-1.10) | 0.6507 | 0.3344 | 17814 | 24135 | *COX11* |
| rs527616 | 18 | 22591422 | C/G | 0.95 (0.93–0.97) | 0.3393 | 0.3468 | 0.7824 | 0.90 (0.79-1.02) | 0.1118 | 0.4218 | 49671 | 67296 | *18q11.2* |
| rs1436904 | 18 | 22824665 | G/T | 0.96 (0.94–0.98) | 0.3928 | 0.3993 | 0.9929 | 0.98 (0.88-1.10) | 0.7861 | 0.6737 | 73624 | 99749 | *CHST9* |
| rs8170 | 19 | 17250704 | A/G | 1.03 (0.98-1.08) | 0.1404 | 0.1413 | 0.5704 | 1.00 (0.81-1.23) | 0.9896 | 0.791 | 273621 | 370714 | *MERIT40* |
| rs4808801 | 19 | 18432141 | G/A | 0.93 (0.91–0.95) | 0.2843 | 0.2818 | 0.9994 | 0.95 (0.84-1.07) | 0.4018 | 0.7277 | 27792 | 37654 | *SSBP4-ISYNA1-ELL* |
| rs3760982 | 19 | 48978353 | A/G | 1.06 (1.04–1.08) | 0.3712 | 0.3571 | 0.9915 | 1.09 (0.98-1.22) | 0.1242 | 0.6191 | 36951 | 50062 | *C19orf61-KCNN4-LYPD5-ZNF283* |
| rs2284378 | 20 | 32051756 | C/T | 1.05 (1.01-1.10) | 0.7521 | 0.7709 | 0.9954 | 0.99 (0.88-1.13) | 0.9289 | 0.4285 | 68635 | 92989 | *RALY* |
| rs2823093 | 21 | 15442703 | A/G | 0.92 (0.88-0.96) | 0.2587 | 0.2673 | 0.9434 | 0.98 (0.86-1.11) | 0.7159 | 0.3862 | 22101 | 29943 | *NRIP1* |
| rs132390 | 22 | 27951477 | C/T | 1.12 (1.07–1.18) | 0.0187 | 0.0163 | 0.2002 | 1.79 (0.69-4.60) | 0.2259 | 0.3347 | 127133 | 172245 | *EMID1-RHBDD3-EWSR1* |
| rs6001930 | 22 | 39206180 | C/T | 1.12 (1.09–1.16) | 0.061 | 0.0547 | 0.956 | 1.05 (0.83-1.33) | 0.7078 | 0.5764 | 40004 | 54199 | *MKL1* |

*The table describes association at the 72 BC associated SNPs.  For each SNP, we reported the genomic position, the alleles, the OR (and CI) estimated by the BC consortium [7;* [*http://gameon.dfci.harvard.edu/gameon*](http://gameon.dfci.harvard.edu/gameon)*], the frequency in Sardinian cases and controls, the imputation accuracy, the OR and p-value in Sardinians, the heterogeneity between the Sardinian OR and the OR previously reported, the number of samples required to observe each locus with significance <10^-6^ or <5x10^-8^  considering the Sardinian allele frequencies, and the candidate genes in the region.*

**Table S5**. Gender-based allele frequency for the most significant SNPs reported in Table 2.

| **Chromosomal region** | **SNP** | **Position** | **Alleles** | **Freq females** | **Freq males** | ***Pvalue*** | **Gene** |
| --- | --- | --- | --- | --- | --- | --- | --- |
|  |  |  |  |  |  |  |  |
| 1p35.1 | rs9425977 | 34238042 | C/T | 0,0571 | 0,06 | 0,6078 | *CSMD2* |
| 1p34.1 | rs2477618 | 44271050 | C/G | 0,0548 | 0,0586 | 0,5178 | *-* |
| 1p13.3 | rs345299 | 108249656 | A/C | 0,5331 | 0,5338 | 0,7214 | *VAV3* |
| 1q32.1 | rs6661074 | 203605742 | A/G | 0,4078 | 0,4319 | 0,1508 | *LEMD1* |
| 2p25.3 | rs13393791 | 3239073 | C/T | 0,0413 | 0,0375 | 0,514 | *TSSC1* |
| 2p21 | rs17032957 | 45134208 | A/G | 0,7618 | 0,7398 | 0,04413 | *SIX2/SIX3* |
| 2q37.1 | rs838436 | 232291757 | A/G | 0,9235 | 0,9271 | 0,5205 | *-* |
| 3p14.1 | rs9810816 | 67158548 | C/G | 0,9564 | 0,9537 | 0,5002 | *-* |
| 5q35.2 | rs17076993 | 173564400 | C/T | 0,0519 | 0,0471 | 0,4245 | *-* |
| 6q23.1 | rs3777428 | 130495535 | C/T | 0,9591 | 0,9483 | 0,1217 | *L3MBTL3* |
| 7p14.2 | rs6968002 | 36531229 | C/T | 0,9589 | 0,9579 | 0,7115 | *AOAH* |
| 8q24.3 | rs11785598 | 144537061 | C/T | 0,9275 | 0,9297 | 0,7016 | *RHPN1* |
| 9q22.32 | rs10512243 | 97815280 | A/G | 0,0552 | 0,0569 | 0,7817 | *-* |
| 9q31.2 | rs10979327 | 110160516 | G/T | 0,0547 | 0,0628 | 0,2803 | *-* |
| 9q33.3 | rs1928482 | 125472269 | C/T | 0,2978 | 0,3052 | 0,803 | *DENND1A* |
| 9q34.13 | rs1633769 | 134869098 | C/G | 0,8092 | 0,8009 | 0,3415 | *-* |
| 9q34.3 | rs3811159 | 136828478 | A/G | 0,6818 | 0,6519 | 0,04241 | *COL5A1* |
| 10q21.1 | rs1903974 | 53549083 | A/G | 0,0815 | 0,0756 | 0,4037 | *PRKG1* |
| 11p12 | rs7947387 | 38902742 | A/G | 0,0696 | 0,0689 | 0,9329 | *-* |
| 12q21.1 | rs11178748 | 70021794 | A/G | 0,0798 | 0,0779 | 0,5374 | *TSPAN8* |
| 12q23.3 | rs7488485 | 105789959 | C/T | 0,0776 | 0,0712 | 0,2614 | *RIC8B* |
| 13q21.1 | rs9569528 | 56234110 | C/T | 0,0462 | 0,053 | 0,1351 | *-* |
| 14q23.1 | rs17097373 | 59896563 | C/T | 0,0571 | 0,0578 | 0,7932 | *-* |
| 15q12 | rs17651375 | 25763160 | C/T | 0,0533 | 0,0578 | 0,6922 | *OCA2* |
| 16q12.1 | rs2193094 | 51123955 | G/T | 0,477 | 0,4781 | 0,9116 | *TOX3* |
| 16q21 | rs8045513 | 60720397 | A/T | 0,0902 | 0,0867 | 0,6991 | *-* |
| 18p11.22 | rs17436811 | 10080771 | C/T | 0,0565 | 0,0584 | 0,8271 | *-* |
| 19q13.31 | rs17725531 | 49018018 | G/T | 0,0823 | 0,1065 | 0,0256 | *-* |
| 20p12.2 | rs6039942 | 10376146 | A/G | 0,0712 | 0,0667 | 0,3789 | *C20orf94* |
| 21q22.11 | rs2833424 | 31774071 | A/G | 0,2668 | 0,2268 | 0,01246 | *TIAM1* |
| 21q22.11 | rs963950 | 33438577 | C/T | 0,8943 | 0,8787 | 0,2205 | *IFNAR2* |
| 21q22.13 | rs857989 | 38042001 | C/G | 0,1 | 0,1157 | 0,1258 | *KCNJ6* |
| 22q12.3 | rs6000351 | 35340813 | A/G | 0,0678 | 0,0701 | 0,7574 | *CACNG2* |
